# AKR1B8 deficiency drives severe DSS-induced acute colitis through invasion of luminal bacteria and activation of innate immunity

**DOI:** 10.3389/fimmu.2022.1042549

**Published:** 2022-11-28

**Authors:** Qiulin Deng, Yichen Yao, Jing Yang, Ramina Khoshaba, Yi Shen, Xin Wang, Deliang Cao

**Affiliations:** ^1^ Department of Proctology, The Affiliated Nanhua Hospital, University of South China Hengyang Medical School, Hengyang, Hunan, China; ^2^ Hospital of Stomatology, Guanghua School of Stomatology, Sun Yat-sen University and Guangdong Provincial Key Laboratory of Stomatology, Guangzhou, China; ^3^ Department of Gastroenterology, The First Affiliated Hospital, University of South China Hengyang Medical School, Hengyang, Hunan, China; ^4^ Department of Medical Microbiology, Immunology, and Cell Biology, Southern Illinois University, School of Medicine, Springfield, IL, United States; ^5^ Department of Biotechnology, College of Science, University of Baghdad, Baghdad, Iraq; ^6^ Department of Medicine, Harvard Medical School, Boston, MA, United States; ^7^ Jeff and Penny Vinik Center for Translational Immunology Research, Division of Allergy and Clinical Immunology, Brigham and Women’s Hospital, Boston, MA, United States

**Keywords:** AKR1B8, acute colitis, pathogenesis, innate immunity, luminal bacteria

## Abstract

**Background:**

Dysfunction of intestinal epithelial cells (IECs) promotes inflammatory bowel disease (IBD) and associated colorectal cancer (CRC). AKR1B8 deficiency impairs the IEC barrier function, leading to susceptibility to chronic colitis induced by dextran sulfate sodium (DSS), yet it remains unclear how acute colitic response is in AKR1B8 deficient mice.

**Methods:**

AKR1B8 knockout (KO) and littermate wild type mice were exposed to oral 1.5% DSS in drinking water for 6 days. Disease activity index and histopathological inflammation scores by H&E staining were calculated for colitic severity; permeability was assessed by fluorescein isothiocyanate dextran (FITC-Dextran) probes and bacterial invasion and transmission were detected by *in situ* hybridization in mucosa or by culture in blood agar plates. Immunofluorescent staining and flow cytometry were applied for immune cell quantification. Toll-like receptor 4 (TLR4) and target gene expression was analyzed by Western blotting and qRT-PCR.

**Results:**

AKR1B8 KO mice developed severe acute colitis at a low dose (1.5%) of DSS in drinking water compared to wild type controls. In AKR1B8 KO mice, FITC-dextran was penetrated easily and luminal bacteria invaded to the surface of IEC layer on day 3, and excessive bacteria translocated into the colonic mucosa, mesenteric lymph nodes (MLNs) and liver on day 6, which was much mild in wild type mice. Hyper-infiltration of neutrophils and basophils occurred in AKR1B8 KO mice, and monocytes in spleen and macrophages in colonic mucosa increased markedly compared to wild type mice. TLR4 signaling in colonic epithelial cells of AKR1B8 KO mice was activated to promote great IL-1β and IL-6 expression compared to wild type mice.

**Conclusions:**

AKR1B8 deficiency in IECs drives severe acute colitis induced by DSS at a low dose through activation of the innate immunity, being a novel pathogenic factor of colitis.

## Introduction

Ulcerative colitis (UC) is a type of chronic inflammatory bowel disease (IBD) (1). The exact etiopathogenesis of UC is unclear, but growing evidence suggests that abnormal intestinal barrier function promotes UC pathogenesis (2), which includes increased intestinal epithelial permeability, reduced mucus production, and defects in cell integrity (3). Intestinal epithelial cells (IECs) are the single cell layer that are constantly renewed under rigid regulation effectively separating luminal pathogens from the lamina propria. IEC dysfunction may disrupt intestinal barrier function and induce colitis or colitis susceptibility (4–7).

The intestinal microenvironment contains IECs, immune cells, and gut microbiota; the interaction among IECs, gut bacteria, and immune cells is complex and maintained in a dynamic homeostasis, and deregulation of any components may cause intestinal diseases (8, 9). Dextran sulfate sodium (DSS) is a wildly used chemical for induction of colitis in mice, which imitates clinical human colitis (10, 11). DSS could cross the mucus layer and cause damage to the IECs, thus allowing the penetration of luminal bacteria and bacterial products into the submucosa and deep tissues (12), which activates innate and adaptive immunity and promotes mucosal pathogenesis (13).

Aldo-keto reductase 1B10 (AKR1B10) is a monomeric cytosolic enzyme with activity to toxic α, β-unsaturated carbonyl compounds, thus protecting host cells from DNA damage (14). AKR1B10 is primarily expressed in IECs of colon and small intestine in healthy humans (15), while AKR1B10 protein is downregulated or lost in patients with IBD and associated CRC, being a potential pathogenic factor (16). Furthermore, AKR1B10 mediates long chain fatty acid and thus lipid synthesis *via* regulating acetyl-CoA carboxylase-α (ACCα) stability (17), through which AKR1B10 promotes the synthesis of membrane phosphatidylinositol 4,5-bisphosphate (PIP_2_) (18). PIP_2_ is a critical signal molecule that regulates membrane-based signaling transduction (19).

To date, it is unknown how AKR1B10 is involved in the pathogenesis of IBD. Thus, we generated aldo-keto reductase 1B8 (AKR1B8, the ortholog of human AKR1B10) knockout mice to explore its potential etiopathological role in colitis. We previously showed that AKR1B8 has a similar tissue distribution pattern and function to AKR1B10 (20). AKR1B8 regulates IEC barrier functions and intestinal immunity (21), and loss of AKR1B8 causes impaired IEC renewal and increased chronic colitis susceptibility (16). However, it is unclear of the effect of AKR1B8 deficiency on acute response to DSS exposure. In this report, we demonstrated that AKR1B8 KO mice developed severe acute colitis under 1.5% DSS treatment. FITC-dextran permeability robustly increased; luminal bacteria reached the surface of IECs at day 3, and then penetrated into submucosa, MLNs, and liver at day 6. Meanwhile, neutrophils, macrophages and basophils exclusively infiltrated and TLR4 signaling was activated in the colon of AKR1B8 deficient mice with acute colitis induced by DSS at 1.5%.

## Methods

### Animals

Mice were housed in the animal facility at University of South China at 24°C ± 0.5°C, 50% ± 10% humidity with 12 hours of light from 8:00 am to 8:00 pm and free access to regular commercial diet and tap water. Heterozygous AKR1B8 knockout (+/-) C57BL/6 mice were used to produce homozygous AKR1B8 knockout (-/-) and littermate wild type (+/+) mice for experimental studies.

### DSS treatments

For acute colitis induction, mice (9 to 10 weeks old) were administered 1.5% dextran sulfate sodium (DSS, molecular weight 40,000 kDa; Gojira Fine Chemicals, LLC) for 6 days. Animals were monitored daily for body weight, occult and gross rectal bleeding, and diarrhea for disease activity. At indicated time points, mice were euthanized and the entire colon was excised, measured, and embedded with paraffin using a standard procedure or subjected to cell isolation (16).

### *In vivo* permeability assays

Intestinal permeability was measured by oral trace amount of FITC-dextran (4000 MW; TdB Consultancy) (0.5 g/kg body weight) for 6 days. At indicated time points, mice were euthanized; colon, MLNs and livers were excised and embedded with paraffin using a standard procedure. Green fluorescence was quantitated with ImageJ software.

### Immunofluorescent staining and histological assays

Colon tissue were harvested and fixed with 4% PFA overnight, after PBS washing for 3 times, paraffin embedding was performed. Sections with 6 µm thickness were prepared. Slides were incubated with the FITC-labeled anti-Muc2 (Sigma) antibody overnight, wash with PBS and co-stained with DAPI, then mounted with Fluoromount (Sigma). H&E staining were performed using standard (21).

### Disease activity index

Disease activity index (DAI) was calculated as previous described (16). Briefly, this scoring system assesses three variables, including body weight, stool consistency, and gross blood. Weight changes are indicative of colitis severity and were monitored every other day. Fecal shape/diarrhea and inflammation-related gross bleeding were recorded. Occult bleeding was measured by using a fecal occult blood test kit (Hemoccult SENSA; Beckman counter, CA).

### Histopathological inflammatory scores

Inflammation induced by DSS was evaluated as previously reported (16). Severity of inflammatory histological alterations in the colon mucosa were graded from 0 to 4. Severity of epithelial damage, infiltration, and lesions were calculated.

### Epithelial crypts and lamina propria leucocyte isolation

Colonic epithelial crypts and lamina propria cells were isolated as previously reported (22). Briefly, the colon was cut into small pieces and shaken twice for 20 min each in HBSS buffer supplemented with 2% FBS, 5 mM EDTA and 1 mM DTT. Epithelial cryps were collected at the bottom and purified in gradient Percoll. The remainder of the tissues were cut and incubated in a shaking incubator in Dulbecco’s PBS containing 10% FBS, 0.5 mg/ml collagenase D (Roche), 0.5 mg/ml dispase II (Roche) and 100 U DNase I (Sigma) for two consecutive 20 min at 37˚C. After digestion, the suspensions were collected and enriched in gradient Percoll.

### Cells isolation from MLN and spleen

MLNs and spleens were collected, cut into small pieces in 40μm nylon cell strainer, and then squeezed with syringe tips. Single cell suspensions were collected from the flow-through of 40μm nylon cell strainer. For spleen cell isolation, RBC lysis buffer (Biolegend) was used for blood cell exclusion according to the manufacturer’s instructions.

### Flow cytometry

After blocking the Fc receptors, cells were stained with appropriate cell surface marker antibodies for 20 min. For intracellular staining, cells were stimulated for 5hrs in 1640 medium containing 10% FBS, 50 ng/ml Phorbol 12-Myristate 13-Acetate (PMA) and 500 ng/ml ionomycin (Sigma) in the presence of GolgiStop (BD). CD11c (N418), Ly6G (1A8), Ly6C (HK1.4), CD45 (30-F11), c-kit (2B8) and FcϵRI (MAR-1) antibodies were used for staining of specific cell markers. 7-amino-actinomycin D (7AAD) was used for dead cell exclusion. All antibodies and reagents were purchased from Biolegend or BD. Flow cytometry analysis of stained cells was performed using an Accuri C6 flow cytometer, and data were analyzed using FlowJo software (Tree Star).

### Fluorescent *in situ* hybridization

Frozen sections with 8µm were prepared. The tissue sections were incubated with 800 ng Alexa Fluor 555-conjugated EUB (5’-GCTGCCTCCCGTAGGAGT-3’) (bp 337-354 in bacteria EU622773) (23) in 50 μl of hybridization buffer (20 mM Tris-HCl (pH 7.4), 0.9M NaCl, 0.1% SDS) at 50°C overnight. The sections were rinsed in wash buffer (20 mM Tris-HCl (pH 7.4), 0.9 M NaCl], washed at 50°C for 20 min, Co-stained with anti-muc2 (Sigma) and DAPI were performed at 4°C and mounted with Fluoromount (F4680, Sigma).

### Bacterial cultures

MLNs and liver were removed, weighted and homogenized with a sterile grinder, and then serial dilutions of bacterial supernatants were plated onto blood agar (Sigma). Cultures were incubated at 37°C and examined overnight. Bacterial counts on the blood agar plates were quantified as the number of colony-forming units (CUF) per microgram of tissues.

### Real-time RT-PCR

Total RNA was extracted from colonic epithelial crypts using TRIzol (Invitrogen). 1μg of RNA was used for reverse transcription. The cDNA products were used as templates for real-time RT-PCR with SYBR green qPCR mixture following the manufacturer’s protocol. Reactions were run on the 7500 Real-Time PCR System (Applied Biosystems). Data were normalized to β-actin and relative mRNA expression was determined using the ΔΔCt (24). Gene-specific primer sequences for IL1b:

Forward GCAACTGTT CCT GAA CTC AAC
Reverse ATC TTT TGG GGT CCG TCA ACT.

IL6:

Forward TAGTCCTTCCTACCCCAATTTCC
Reverse TTGGTCCTTAGCCACTCCTTC.

β-actin

Forward CGGTTCCGATGCCCTGAGGCTCTT
Reverse CGTCACACTTCATGATGGAATTGA

### Western blotting

Colonic epithelial crypts were homogenized in lysis buffer (Roche), followed by centrifugation at 4°C, 14,000 *g* for 5 minutes. Soluble proteins were separated on SDS-PAGE and then blotted onto a nitrocellulose membrane (Bio-Rad). The membrane was incubated with the anti-TLR4 antibody (Santa Cruz), followed by a secondary antibody (LI-COR). Antibody binding was detected by Odyssey Infrared Imaging System (LI-COR). Protein amount was adjusted by β-actin antibody (Sigma).

### Statistical analysis

Prism 4 (Graph Pad) was applied for statistical analysis. Variance test, Student t-test, or one-way ANOVA test were used to compare the differences between AKR1B8 KO and wild type mice, with p < 0.05 as statistical significance.

## Results

### AKR1B8 deficient mice develop severe acute colitis induced by a low dose of DSS

**Figure 1:**
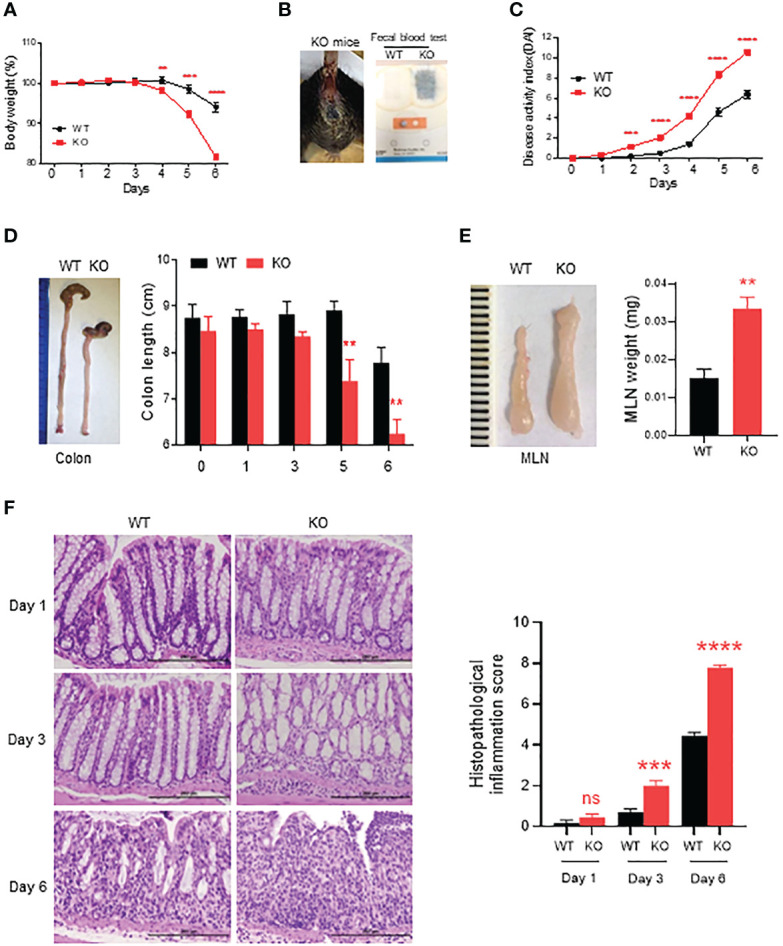
Severe acute colitis induced by DSS in AKR1B8 deficient mice. AKR1B8 KO and wild type mice were treated with 1.5% DSS in drinking water for 6 days. **(A)** Body weight measured daily. **(B)** Bleeding in AKR1B8 KO mice. *Right panel*, fecal occult blood tests. **(C)** Disease activity index (DAI). **(D)** Colon length on day 6. **(E)** Mesenteric lymph nodes (MLNs) on day 6 (n=6). **(F)** Histological assessment of colonic tissues during the course (day1, day3 and day6) of acute colitis induction. *Right panel*, histopathological inflammation. Scale bar, 200μm. KO, knockout; WT, wild type. **p < 0.01; ***p < 0.001 and ****p < 0.0001; ns, No Significance.

DSS is a chemical widely used for induction of colitis in mice. In AKR1B8 deficient mice, chronic feeding of DSS in drinking water for 4 cycles induced severe colitis and associated tumorigenesis (16). This study evaluated the acute response of AKR1B8 deficient mice to a low dose of DSS at 1.5% in drinking water for 6 days. As shown in **Figure 1A**, AKR1B8 KO mice demonstrated body weight loss starting at day 4 and peaked up to 20% body weight loss at day 6 compared to only 4% weight loss in wild type (WT) mice. Severe diarrhea and gross bleeding appeared in AKR1B8 KO mice at day 6, but not in wild type mice (**Figure 1B**). The disease activity index (DAI) was significantly higher in AKR1B8 KO mice than in wild type starting at day 2 (**Figure 1C**). At the gross histological level, colon length significantly decreased after 5 days of DSS administration in drinking water (**Figure 1D**), accompanied with enlarged MLNs (**Figure 1E**) in AKR1B8 KO mice compared to wild type controls. No clear differences were noted in size and weight of spleens between AKR1B8 KO and wild type mice (data not shown).

We further assessed histopathology of the colon over the course of acute colitis induced by DSS at 1.5%. On day 1 of DSS exposure, the histological structure of colon mucosa was normal, but mild infiltration of inflammatory cells appeared in KO mice. Otherwise, no clear differences were noted between AKR1B8 KO and wild type mice (**Figure 1F**, *left upper panel*). However, heavy infiltration of leukocytes and swelling occurred in colonic mucosa of AKR1B8 KO mice on day 3 of DSS exposures (**Figure 1F**, *left middle panel*). On day 6, dramatic destruction of histological structures appeared in the colonic mucosa of AKR1B8 KO mice but not in that of wild type mice (**Figure 1F**, *left lower panel*). Ulceration of mucosa occurred in AKR1B8 KO mice, but not in wild type mice, indicating the severe mucosal injury. The histological inflammation scores, as determined by quantitative epithelial damage and inflammatory cell infiltration, were significantly higher in AKR1B8 KO mice than in wild type mice (**Figure 1F**, *right panel*). Taken together, these data indicate that AKR1B8 deficiency leads to much more severe acute colitis induced by a low dose of DSS in drinking water.

### Severe infiltration of innate immune cells in the colon mucosa of AKR1B8 deficient mice exposed to a low dose of DSS

**Figure 2:**
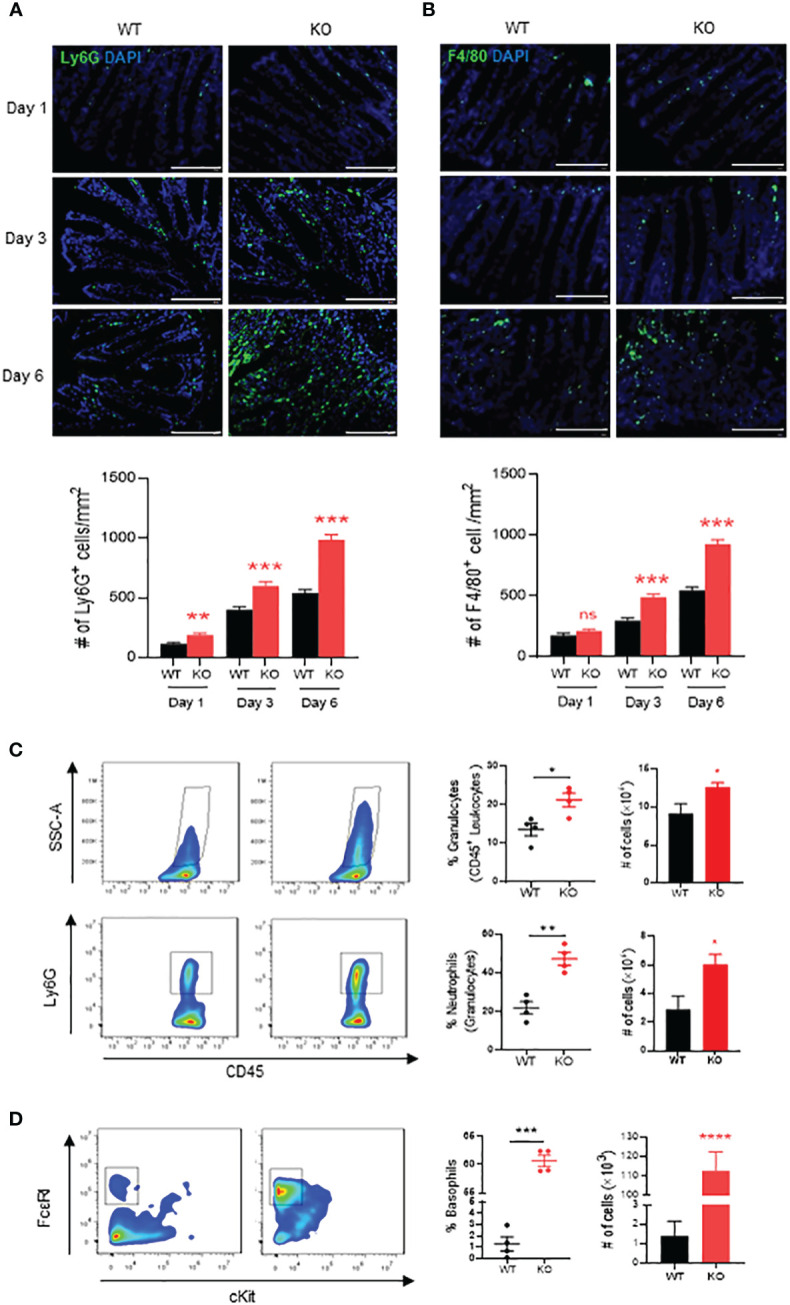
Neutrophils, macrophages and basophils in colon of AKR1B8 deficient mice under acute colitis. Immunofluorescent staining was used to evaluate innate immune cells in the colon mucosa during the course of acute colitis induced by DSS at 1.5%. **(A)** Ly6G^+^ neutrophils. **(B)** F4/80^+^ macrophage. *Lower panel*, Ly6G^+^ and F4/80^+^ cell number per mm^2^. Scale bar, 100μm. Thereafter, flow cytometry was used to confirm the innate immune cell changes as described in Materials and Methods. **(C)** granulocytes (SSC-A^high^ CD45^+^; *upper panel*) and neutrophils (SSC-A^high^ CD45^+^ Ly6G^+^; *lower panel*). **(D)** basophils (SSC-A^high^ CD45^+^ c-Kit^-^ FCϵRI^+^). From *left* to *right*, flow cytometry images, percentage and absolute number of cells. KO, knockout; WT, wild type. *p < 0,05; **p < 0.01; ***p < 0.001 and ****p < 0.0001; ns, No Significance.

We then investigated innate immune cells infiltrated in the inflammatory mucosa of AKR1B8 KO mice exposed to 1.5% DSS. Neutrophils and macrophages are major effector cells for acute inflammation and are the first responders in the intestinal inflammation for elimination of invaded pathogens (25). Therefore, we assessed the neutrophils and macrophages in DSS-induced colitic mucosa on days 1, 3 and 6. As shown by immunofluorescent (IF) staining with a Ly6G antibody, infiltration of Ly6G^+^ neutrophils started at day 1 of DSS exposure, which increased at day 3 and then robust at day 6 (**Figure 2A**). F4/80^+^ macrophages also increased in the mucosa of colon and accumulated at day 3 of DSS treatment and severely peaked up at day 6 (**Figure 2B**).

**Figure 3:**
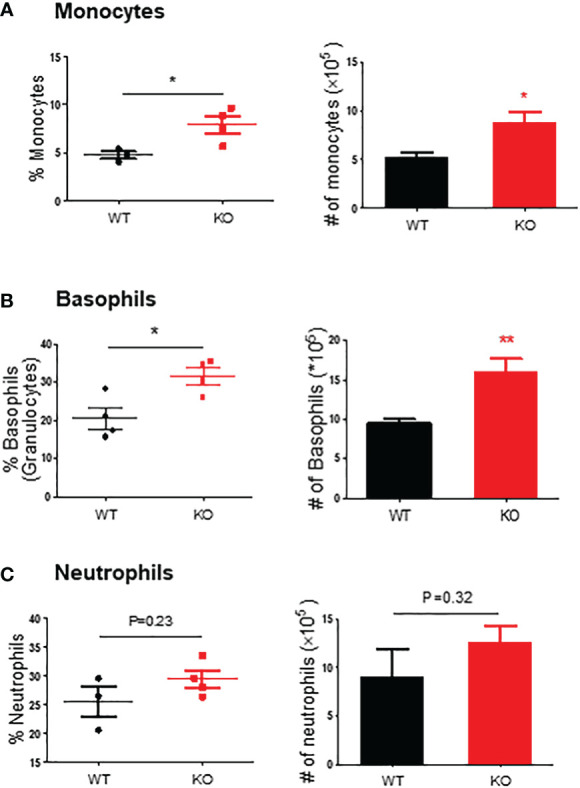
Monocytes, basophils, and neutrophils in the spleen of mice exposed to DSS. Flow cytometry was used to assess monocytes (SSC-A^high^ CD45^+^ Ly6C^+^ Ly6G^-^) **(A)**, basophils (SSC-A^high^ CD45^+^ c-Kit^-^ FCϵRI^+^) **(B)**, and neutrophils (SSC-A^high^ CD45^+^ Ly6G^+^) **(C)** in the spleen of mice exposed to DSS at 1.5%, as described in the Materials and Methods. *Left panel*, percentage; *right panel*, absolute number of cells. KO, knockout; WT, wild type. *p < 0,05; **p < 0.01.

We further evaluated the innate immune cell infiltrations by flow cytometry on day 6. The results showed that in the colon mucosa of AKR1B8 KO mice, CD45^+^FSC^hi^ granulocytes significantly increased when compared to wild type mice (**Figure 2C**, *upper panel*), and Ly6G^+^ neutrophils increased by nearly 3 folds (**Figure 2C**, *lower panel*). These data confirmed the hyper-infiltration of neutrophils in the acute colitis of AKR1B8 KO mice induced by a low dose of DSS. Surprisingly, cKit^-^ FcϵRI^+^ basophils in the acute colitis exclusively increased up to 58.1% in AKR1B10 KO mice vs. 1.4% in wild type (**Figure 2D**); the absolute number of cKit^-^ FcϵRI^+^ basophils increased over 100 times in AKR1B10 KO mice compared to wild type (**Figure 2D**, *right panel*)). These data indicate the dramatic infiltration of inflammatory cells in DSS-induced colitis of AKR1B8 KO mice, which is consistent with the severe inflammatory response observed. We also measured neutrophils, monocytes and basophils in the spleen of DSS-exposed mice, and the results showed that the monocytes and basophils also significantly increased in the spleen of AKR1B8 KO mice compared to that of wild type (**Figures 3A, B**); the neutrophils were tended to be elevated in the spleen of AKR1B8 KO mice exposed to DSS at 1.5%, but not statistically significant (**Figure 3C**). These innate cell changes in the spleen of AKR1B8 mice seem consistent with those in the mucosa.

### FITC-dextran penetration into the intestinal mucosa, MLN and liver of AKR1B8 deficient mice exposed to a low dose of DSS

**Figure 4:**
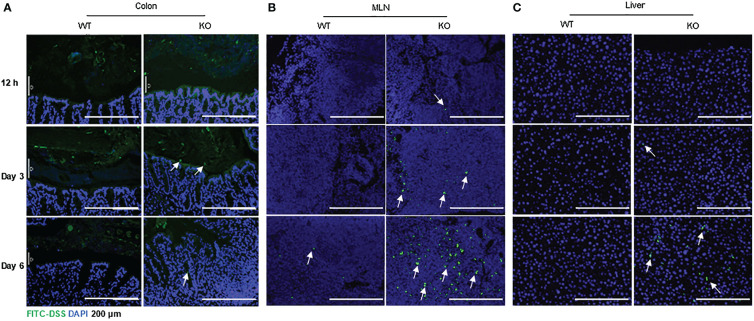
Increased FITC-DSS penetration into colon, MLN and liver of AKR1B8 KO mice. FITC-labeled dextran was administered and its penetration into the organs was evaluated as described in the Materials and Methods. Nuclei were stained with DAPI (Blue). **(A)** colonic mucosa, **(B)** Mesenteric lymph nodes (MLNs), and **(C)** liver. Arrows indicate the FITC-dextran. Scale bars, 200μm. KO, knockout; WT, wild type.

IECs are critical for gut health, and IEC dysfunction may cause barrier defects and induce colitis or colitis susceptibility (2, 8). With the severe DSS-induced colitis in AKR1B8 KO mice, we further investigated the integrity of colonic epithelium with FITC-labeled dextran. Mice were fed with FITC-dextran (0.5 g/kg body weight) by oral gavage, and the tissue samples were collected at indicated time points. As shown in **Figure 4A**, FITC-dextran was restricted in the lumen of both wild type and AKR1B8 KO mice at Day 1 of exposure, but FITC-dextran free mucus layer was thinner in AKR1B8 KO mice at Day 3, and FITC-dextran diffused into the mucus and reached the epithelial surface in AKR1B8 KO mice, but not in wild type. At Day 6 of exposure, FITC-dextran penetrated into the colonic mucosa of AKR1B8 KO mice, but was mainly restricted in the lumen in wild type mice, where an integrated mucus layer appeared, separating the FITC-dextran from colonic epithelium. Consistently, FITC-labeled dextran appeared in the MLNs of AKR1B8 KO mice at day 1 of exposure and accumulated at day 3 and robustly at day 6 (**Figure 4B**). No FITC-dextran was observed in the MLNs of wild type mice at day 1 and day 3 of exposures, but a few FITC-dextran signals were detected in the MLNs of wild type mice at day 6 (**Figure 4B**). FITC-labeled dextran was further observed in the liver of AKR1B8 KO mice at day 3 of exposure and dramatically increased at day 6, while no FITC-Dextran was detected in the liver of wild type mice even at day 6 (**Figure 4C**). These data suggest the severe damage of epithelial mucosa in its histology and barrier function in the AKR1B8 deficient mice.

### Dramatic bacterial invasion into the colonic mucosa, MLNs and liver of AKR1B8 deficient mice exposed to a low dose of DSS

**Figure 5:**
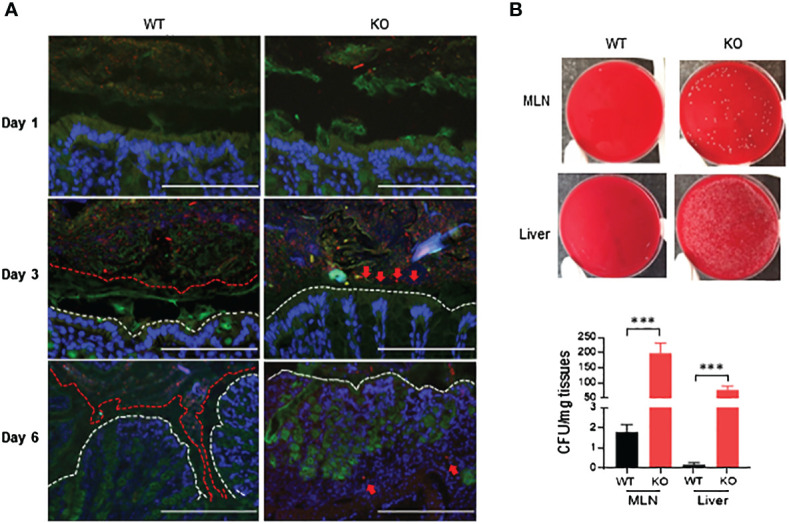
Luminal bacterial invasion in colon and dissemination into mesenteric lymph nodes and live of AKR1B8 KO mice under acute colitis. **(A)** Bacterial invasion in the colon. Bacteria were stained by fluorescent *in-situ* hybridization using the general bacterial rRNA probe, EUB338 conjugated with Alxea 555 (red). The mucus was visualized by immunostaining of the same section with an anti-Muc2 specific antibody (green). White dotted line indicates the colonic epithelium. Arrows point out the penetration of bacteria into inner layer of mucus (day 3) and colonic lamina propria (day 6) in AKR1B8 KO mice in DSS treatment. Scale bars, 200μm. **(B)** Bacterial dissemination into MLN and liver. Suspension (supernatant) from mesenteric lymph nodes (MLN) and liver was subjected to bacterial cultures in blood agar plates on day 6 (*upper panel*), and the numbers of bacterial cfu were counted (*lower panel*). KO, knockout; WT, wild type. ***p < 0.001.

Growing evidence suggests that luminal pathogenic bacteria contribute to pathogenesis of colitis (26, 27) and colorectal cancer development (28). We investigated potential bacterial invasion into colonic mucosa using fluorescence *in-situ* hybridization (FISH) with bacterial 16S rRNA probes. At day 1 of 1.5% DSS exposure, bacteria were restricted in the lumen in both AKR1B8 KO and wild type mice (**Figure 5A**, *upper panel*). At day 3 of DSS treatment, bacteria invaded into the mucus layer and reached the epithelium in AKR1B8 KO mice, but not in wild type mice (**Figure 5A**, *middle panel*). At day 6, the mucus layer disappeared, and bacteria invaded into the colonic mucosa of AKR1B8 KO mice; in wild type mice, however, the mucus layer narrowed, but was still visible, and bacterial invasion into mucosa was not markedly noted (**Figure 5A**, *lower panel*).

MLNs are secondary lymphoid organs and active sites for luminal bacterial antigen presentation. We evaluated the invasion of bacteria into the MLNs by culturing in blood agar plates. Data showed that nearly 200 colony-forming units (CFU)/mg MLN cultures appeared in AKR1B8 KO mice in acute colitis on day 6 induced by 1.5% DSS, but much less in wild type mice (**Figure 5B**). Meanwhile, luminal bacteria also translocated greatly into the liver of AKR1B8 KO mice in acute colitis compared to wild type mice (**Figure 5B**). Taken together, these data indicate that in the acute colitis induced by DSS at 1.5%, vast invasion and dissemination of luminal bacteria into the colonic mucosa, lymphoid organs, and liver occurs.

### Activation of TLR4 signaling in the colon of AKR1B8 KO mice in acute colitis induced by DSS

**Figure 6:**
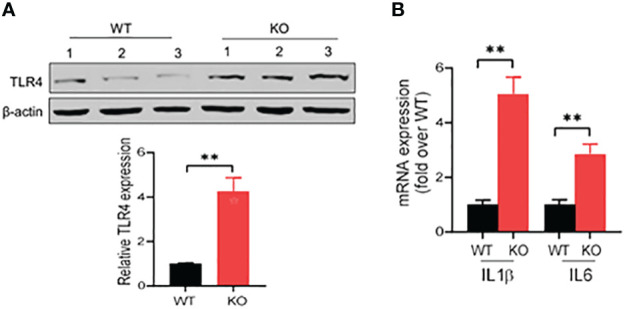
Activation of TLR4 signaling in colon of AKR1B8 KO mice under acute colitis. Colonic mucosa was collected and processed for Western blot and qRT-PCR as described in th Materials and Methods. **(A)** Western blot, demonstrating TLR4 protein expression level in the mucosa under acute colitis on day 6. Relative expression level of TLR4 was calculated using image J software (*Lower panel*). **(B)** Expression levels of IL1β and IL6 in colonic mucosa under acute colitis on day 6. KO, knockout; WT, wild type. **p < 0.01.

IECs are the first line of host defense and are critical for protection of the host from pathogen invasion and inflammatory lesions. In acute colitis, bacterial antigens, like lipopolysaccharide (LPS), activate the inflammatory signaling cascades in IECs, such as toll-like receptor 4 (TLR4) signaling, promoting production of various inflammatory cytokines and inflammatory responses (29–31). We then investigated the expression of TLR4 in the colonic epithelial cells in acute colitis induced by DSS at 1.5% in drinking water. Our results showed that in acute colitis on day 6, TLR4 expression increased by four times in the colonic epithelial cells of AKR1B8 KO mice compared to wild type mice (**Figure 6A**). We then further measured the expression of IL1β and IL6, which are downstream targets of TLR4 signaling and involved in innate immune cell recruitment and induction of inflammation. Consistently, mRNA levels of IL1β and IL6 were significantly upregulated by 5 and 3 folds, respectively, in the colonic epithelial cells of AKR1B8 KO mice (**Figure 6B**). These data indicate that in the colonic epithelial cells of AKR1B8 KO mice exposed to DSS, innate TLR4 signaling is activated, which triggers the expression of inflammatory cytokines IL1β and IL6 and in turn promotes colitis severity.

## Discussion

AKR1B8 in mice is the ortholog of human AKR1B10 (32), and AKR1B10 expression was lost or diminished in the ulcerative colitis tissues, indicating its involvement in colitis (16). Therefore, the focus of this study was to test the response of AKR1B8 KO mice to acute colitis induced by DSS in order to address the potential pathogenic role of AKR1B10 in human colitis.

AKR1B8 KO and wild type mice raised in the same cages were used to induce acute colitis with a low dose of DSS at 1.5%. Starting from Day 1 of DSS exposure, inflammatory index and various histological and cellular responses were monitored, which lasted continuously for 6 days. DSS is a widely used chemical for colitis induction in animals (33). The precise mechanism of colitis induction by DSS is unclear, but studies have shown that DSS could form nanometer-sized vesicles with medium-chain-length fatty acids (MCFAs), which activate innate inflammatory signaling and causes damage to IECs (34). A low dose of DSS broke the integrity of colonic epithelium of AKR1B8 KO mice and induced severe colitis, suggesting their high sensitivity to DSS-induced acute colitis, which is consistent with the DSS-induced chronic colitis in our previous study (16).

The acute colitis induced by DSS is considered to be largely dependent on innate immunity (10) and does not require adaptive T or B cell involvement (35). We did not observe activation of adaptive immunity in this study (data not shown), but robust innate immune cell changes were recorded. Granulocytes, such as neutrophils and basophils, are innate inflammatory immune cells that function to fight against invading microbes, but excessive infiltration of these cells may cause severe inflammation and tissue damage (36–38). Infiltration of neutrophils occurs in patients with IBD (39), and this was also the case in AKR1B8 KO mice. Surprisingly, exclusive hyper-infiltration of basophils (more than forty folds) was observed in the colonic mucosa of AKR1B8 KO mice exposed to DSS at 1.5%. Basophils are inflammation-promoting cells and associated with allergic diseases, such as asthma and allergic rhinitis (40, 41). Therefore, basophils are abnormally elevated in the sites of parasitic infections and allergic reactions and the anticoagulant heparin and the vasodilator histamine in basophils are important promoting factors of allergic responses (37, 42). The AKR1B8 KO mice suffer from severely increased permeability of intestinal mucosa and increased invasion of bacteria in the colonic mucosa, and thus a possible explanation of the elevated basophils in the colonic mucosa of AKR1B8 KO mice may be ascribed to the increased exposures to various luminal antigens.

The robust basophil recruitment in acute colitis in AKR1B8 KO mice is potentially very interesting, since human IBD patients have increased basophils (43–45). Depletion of basophils by diphtheria toxin ameliorates the colonic inflammation in oxazolone-induced colitis in the Mcpt8^DTR^ mice model (43). Various cytokines and chemokines are reported to regulate the recruitment of basophils. For example, previous studies have shown that basophils can be subgrouped into thymic stromal lymphopoietin (TSLP)-elicited basophils that produce IL4 and IL18, and IL3-elicited basophils that produce proteases, histamine, or leukotrienes (46, 47). IL25 and IL33 can also regulate basophil migration and activation by promoting IL-17RB and ST2 receptor expression in basophils (48, 49).. Human basophils express chemokine receptors like CCR1, CCR2, CCR3, CCR5, CXCR1, CXCR2, and CXCR4 (50). CCL1 and CCL2 promote basophil recruitment in skin lesions in patients with systemic lupus erythematosus (51). Patients with IBD have increased CCL2 expression level (52–54), and CCL5 attracts CCR1 and CCR5-expressing inflammatory cells into colon tissue of trinitrobenzene sulphonic acid (TNBS)-induced colitis mice model (55). CCL11, CCL24 and CCL26 also promote basophil recruitment by inducing expression of receptor CCR3 in IBD (56). Basophils can be directly activated by pathogen-associated molecular patterns (PAMPs) and IgE-crosslinking, aggravating inflammation through various mediators, such as the cytokines IL4, IL-13, IL6, TNFα, CCL3, and CCL4, proteases mMCP-8 and mMCP-11, and other mediators like histamine, platelet-activating factor and lipid mediators (57, 58). We previously found that AKR1B8 is involved in the regulation of AKT, ERK, and NF-κB signaling activity (21), and that these signal pathways are crucial for regulating cytokine production (59–61). However, the precise cytokines responsible for basophil infiltration in colons of AKR1B8 KO mice as well as whether this recruitment is due to epithelial cell-intrinsic effects of AKR1B8, is still unknown.

In the intestinal permeability assessment performed with FITC-dextran, the FITC signals were modest in the colonic mucosa, but much stronger in MLN and liver. This may be explained from the anatomical point. While FITC-dextran was delivered orally at trace amount (tracking), modest signals in colonic mucosa are expected as the huge surface area. The MLN is small and drains from the whole colon, thus FITC presenting in MLN provides a concentrating effect. Therefore, it is not unexpected that the MLN has stronger FITC-dextran signals. A similar situation may exist in the liver.

Colitogenic microbiota contributes to the pathogenesis of IBD and associated colorectal cancer (62). Pathogenic bacteria could release toxic metabolites and cause DNA damage which promotes disease development (28). In this study, we observed that in AKR1B8 KO mice, DSS at a low dose of 1.5% accelerated invasive bacteria coming to contact with IECs on day 3, then penetrating into the colonic mucosa, MLNs, and liver on day 6. This uncontrolled bacterial invasion may activate the innate immune cell responses and contribute to the excessive infiltration of neutrophils and basophils in the colon of AKR1B8 KO mice.

IECs express TLRs and thus are critical for recognition of microbial metabolites and pathogens, involved in the regulation of innate immunity and gut microbiota (63). Among various kinds of TLRs, TLR4 is the most studied receptor for recognizing lipopolysaccharide (LPS). Studies have shown that TLR4 signaling is activated during IBD pathogenesis, and the activated TLR4 signaling triggers innate NF-κB signaling and promotes pro-inflammatory cytokine production, contributing to colitis (5, 64). In this study, the increased expression of TLR4 in the IECs of AKR1B8 KO mice indicates the activation of this innate signaling pathway under acute colitis induced by DSS. IL1β and IL6 are inflammatory cytokines regulated by the TLR4 signaling, promoting pathogenesis of IBD (65). The upregulation of these cytokines is thus pathogenic and promotes the severe colitis induced by DSS in the AKR1B8 KO mice.

This study utilizes mice with whole-body knockout of AKR1B8. However, we indeed evaluated the expression of AKR1B8 in the colonic epithelial cells and immune cells in MLNs and found that AKR1B8 is specifically expressed in the colonic epithelial cells, but not in immune cells (21). We also reported that in this AKR1B8 knockout mice, colonic crypts showed abnormal development and injury repair (16). Considering the importance of intestinal epithelial cells in intestinal immunity, therefore, we believe that the functional abnormalities in colon are originated from deficiency of AKR1B8 in the colonic epithelial cells.

In conclusion, this study reports the susceptibility to and severity of acute colitis induced by a low dose of DSS at 1.5% in AKR1B8 deficient mice. The severe acute colitis in AKR1B8 KO mice is featured with high bacterial penetration into mucosa and distant organs, hyper-infiltration of basophils and neutrophils, and activation of innate TLR4 signaling pathway and subsequent production of inflammatory cytokines IL1β and IL6. Our work suggests that AKR1B8, i.e., AKR1B10 in humans, may participate in development and progression of colitis, being a potential target for clinical management of ulcerative colitis.

## Data availability statement

The original contributions presented in the study are included in the article/supplementary material. Further inquiries can be directed to the corresponding authors.

## Ethics statement

The animal study was reviewed and approved by University of South China Laboratory Animal Care and Use Committee.

## Author contributions

XW and DC conceived and designed the experiments. QD, YY, JY, YS and XW performed the experiments. QD, YY and RK analyzed the data and XW wrote the paper. DC funded and supervised the research. All authors contributed to the article and approved the submitted version.

## Funding

This work was supported in part by Natural Science Foundation of Hunan province (2021JJ40483), Natural Science Foundation of Hunan Province (2021JJ40502), the Key Project of Hunan Department of Public Health (202201043124), Hunan Changzhutan National Independent Innovation None Special Project (2018XK2106), and the PhD Scientific Research Start-up Fund of University of South China (200XQD075).

## Conflict of interest

The authors declare that the research was conducted in the absence of any commercial or financial relationships that could be construed as a potential conflict of interest.

## Publisher’s note

All claims expressed in this article are solely those of the authors and do not necessarily represent those of their affiliated organizations, or those of the publisher, the editors and the reviewers. Any product that may be evaluated in this article, or claim that may be made by its manufacturer, is not guaranteed or endorsed by the publisher.
